# Targeted non-invasive brain stimulation boosts attention and modulates contralesional brain networks following right hemisphere stroke

**DOI:** 10.1016/j.nicl.2024.103599

**Published:** 2024-03-30

**Authors:** Elena Olgiati, Ines R. Violante, Shuler Xu, Toby G. Sinclair, Lucia M. Li, Jennifer N. Crow, Marianna E. Kapsetaki, Roberta Calvo, Korina Li, Meenakshi Nayar, Nir Grossman, Maneesh C. Patel, Richard J.S. Wise, Paresh A. Malhotra

**Affiliations:** a Imperial College London, Department of Brain Sciences, UK; b Imperial College Healthcare NHS Trust, UK; c University of Surrey, Department of Psychology, UK; d University College London, UK; e UK Dementia Research Institute Care Research and Technology Centre, Imperial College London and the University of Surrey, London, UK; f UTHealth, Department of Neurobiology and Anatomy, McGovern Medical School, Houston, US

**Keywords:** Stroke, Neglect, Attention, Vigilance, tDCS

## Abstract

Right hemisphere stroke patients frequently present with a combination of lateralised and non-lateralised attentional deficits characteristic of the neglect syndrome. Attentional deficits are associated with poor functional outcome and are challenging to treat, with non-lateralised deficits often persisting into the chronic stage and representing a common complaint among patients and families.

In this study, we investigated the effects of non-invasive brain stimulation on non-lateralised attentional deficits in right-hemispheric stroke. In a randomised double-blind sham-controlled crossover study, twenty-two patients received real and sham transcranial Direct Current Stimulation (tDCS) whilst performing a non-lateralised attentional task. A high definition tDCS montage guided by stimulation modelling was employed to maximise current delivery over the right dorsolateral prefrontal cortex, a key node in the vigilance network. In a parallel study, we examined brain network response to this tDCS montage by carrying out concurrent fMRI during stimulation in healthy participants and patients.

At the group level, stimulation improved target detection in patients, reducing overall error rate when compared with sham stimulation. TDCS boosted performance throughout the duration of the task, with its effects briefly outlasting stimulation cessation. Exploratory lesion analysis indicated that response to stimulation was related to lesion location rather than volume. In particular, reduced stimulation response was associated with damage to the thalamus and postcentral gyrus. Concurrent stimulation-fMRI revealed that tDCS did not affect local connectivity but influenced functional connectivity within large-scale networks in the contralesional hemisphere.

This combined behavioural and functional imaging approach shows that brain stimulation targeted to surviving tissue in the ipsilesional hemisphere improves non-lateralised attentional deficits following stroke. This effect may be exerted via contralesional network effects.

## Highlights

• High-definition non-invasive brain stimulation can be safely used to optimise the delivery of electrical current over spared prefrontal cortex in stroke.
• Precise targeting of prefrontal cortex with tDCS, as compared to sham tDCS, boosts vigilance in patients with right hemispheric stroke.
• Non-responders to the application of tDCS have brain damage centred on the right thalamus and the right postcentral gyrus.
• Performance on a vigilance task depends on the integrity of two large white matter tracts (the right corpus callosum and the right cingulum), suggesting a role of inter-hemispheric and fronto-parietal disconnections in vigilance dysfunction.
• Brain stimulation was associated with widespread fMRI network changes increasing contralateral functional connectivity in stroke patients and age-matched healthy individuals.
• Brain stimulation is a safe treatment that could be integrated into clinical practice as an add-on therapy for patients with attentional impairments.

## 1 Introduction

Following right-hemisphere stroke, patients are frequently affected by the neglect syndrome, which includes lateralised and non-lateralised attentional deficits (Bartolomeo et al., 2012, Corbetta and Shulman, 2011). Neglect is challenging to treat; lateralised deficits, whereby patients ignore targets located on the left hand side of space, most frequently represent the target of therapeutic studies (Harvey et al., 2022, Olgiati and Malhotra, 2020). However, non-lateralised attentional impairments such as deficits of *vigilant attention*, the ability to maintain goal-oriented behaviour over time, tend to persist and are strongly associated with greater functional disability (McDowd et al., 2003, Van Vleet and DeGutis, 2013). At present, there are no convincing randomised trials demonstrating clinically significant improvements in neglect.

The current work was motivated by the potential clinical translation of non-invasive brain stimulation to remediate non-lateralised attentional deficits in patients with right hemisphere damage. Non-invasive brain stimulation using transcranial Direct Current Stimulation (tDCS), which acts by altering neuronal firing rate, has been shown to modulate synaptic efficiency in healthy and stroke populations (Vallar & Bolognini, 2011). Vigilance has consistently been linked to a right-dominant frontoparietal network that includes the right DLPFC, and consistent with this, studies have showed that prefrontal tDCS improves attention in healthy younger and older adults (Brosnan et al., 2018, Hemmerich et al., 2023, Nelson et al., 2014) as well as individuals with traumatic brain injury (Kang, Kim, & Paik, 2012). Two proof-of-principle studies have also shown encouraging results in stroke, but researchers did not specifically test for non-lateralised attention and the number of included stroke patients was relatively small (Kang et al., 2009, Park et al., 2013).

Here we performed a double-blind, sham-controlled crossover study to test the efficacy of tDCS application to surviving ipsilesional tissue in stroke patients. In conventional tDCS, large electrodes (typically two 5x7cm) are positioned far apart on the scalp, resulting in the current travelling long distances and potentially influencing several cortical and subcortical regions (Moreno-Duarte et al., 2014). The high-definition tDCS (HD-tDCS) approach we used utilises denser electrode arrays, increasing precision and optimising targeting of a region of interest by constraining the current to the area between the electrodes (e.g., see Datta et al., 2009).

We hypothesised that *online* tDCS (i.e., stimulation applied during a cognitive task) would lead to an improvement in task performance.

To shed light on the underlying neurophysiological mechanisms of tDCS, we also conducted a parallel tDCS-fMRI study examining functional connectivity changes as patients received brain stimulation at rest while in the MRI scanner, in an *offline* design. We employed resting-state methodology to maximise scanning tolerability, thereby mitigating against over-representing mildly affected patients. Following on from evidence from healthy individuals, we hypothesised that HD-tDCS would modulate connectivity within large-scale attentional networks rather than solely affecting local connectivity (Li et al., 2019).

## 2 Materials and methods

**Fig. 1:**
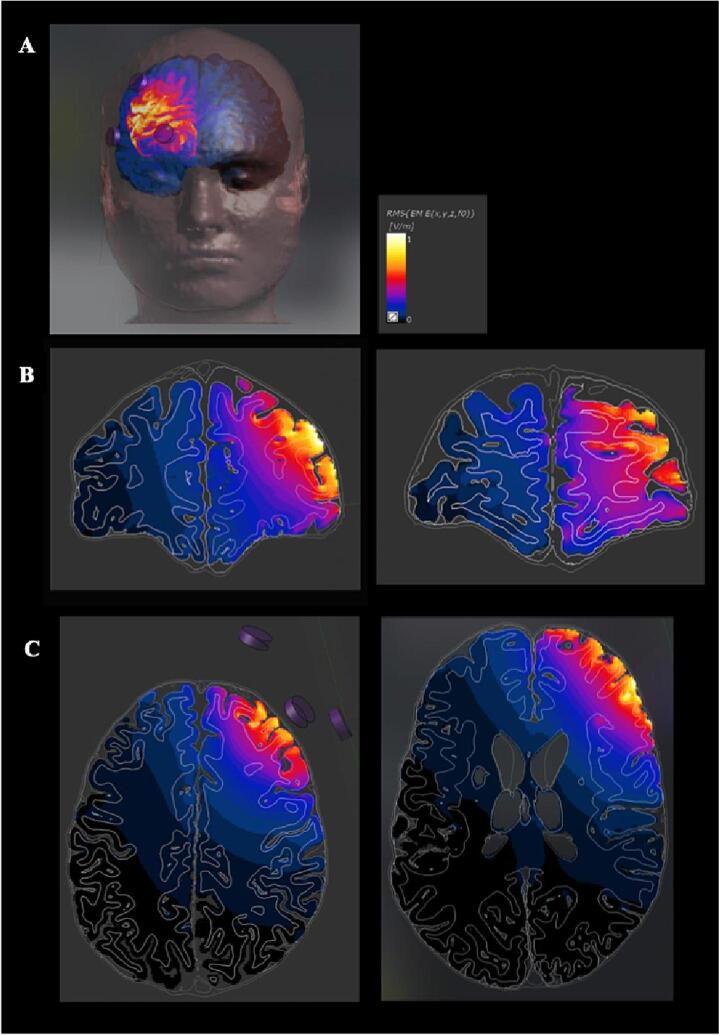
Computational modelling confirming targeted delivery of brain stimulation over the right dorsolateral prefrontal cortex. A. Finite element model of the electrical field distribution, with grey matter superimposed; B and C. Modelling in coronal and axial plane of the electrical field distribution, showing the depth of current penetration and the focality achievable with the tDCS montage used in the present work. Computational modelling allowed us to visualise that the chosen montage led to maximal current density over the right DLPFC, with a superficial cortical passage of current. Clinical brain imaging was inspected prior to recruitment to ensure the DLPFC was spared in our population. Note that this montage is well suited for cortical modulation of a circumscribed superficial cortical area. Model created using Sim4Life, ZurichMedTech, Zurich, Switzerland.

Precise targeting of the DLPFC was achieved through use of a modelled High-Definition (HD) focal montage that constrained current to spared cortex (Bikson et al., 2012, Dmochowski et al., 2013) (Fig. 1) (Supplementary material 3).

To identify individuals likely to have persistent chronic attentional deficits, we screened for neglect on acute stroke wards at Charing Cross Hospital, London, UK. Patients were recruited if they showed evidence of neglect on clinical tests acutely following a first documented unilateral lesion in the right cerebral hemisphere, with no other neurological or psychiatric diagnosis (Table 1, Supplementary material 7). After a minimum of three months post-stroke, i.e., past the peak of spontaneous recovery (Kreisel, Hennerici, & Bäzner, 2007), 41 patients matching standard tDCS and imaging inclusion/exclusion criteria (Supplementary material 1) were contacted to discuss participation in this research. Sample size calculation was based on previous work (Nelson et al., 2014), which gave an estimated sample size of 14 with an estimated effect size of 0.98 (alpha = 0.05; power = 0.9). We set a recruitment target of 18–22 patients per study to account for possible dropouts and censoring criteria. Written informed consent was obtained. Ethical approval was granted by the Central London Research Ethics Committee. The study conformed to the Declaration of Helsinki.

**Table 1:** Clinical presentation of patients who took part in Study 1 (Behavioural study) and Study 2 (Imaging study).

ID	Study	Days from stroke	Extinction	Star cancellation (% total)	Star cancellation (% Left)	Star cancellation (% Right)	Cancellation start	Line Bisection %
1	1 & 2	25	V, T	25	0	50	Right	+1.8
2	1 & 2	51	V	98*	97*	100*	Right	+4
3	1 & 2	12	V, T	0	0	0	Right	+12.16
4	1 & 2	4	T	96*	93* (4 errors)	100* (1 error)	Right	−5.60
5	1	5	NA	48	20	77	Right	+12.60
6	1 & 2	1	V	29	0	58	Right	+85.5
7	1	1	V	84	87	81	Right	+37
8	1 & 2	3	T	98^†^	97^†^	100^†^	Left	+0.8
9	1 & 2	5	V, T	73	93	54	Right	+1.60
10	1 & 2	12	V	79	73	85	Right	+1.83
11	1 & 2	7	V, T	62	10	81	Right	+16.60
12	1 & 2	15	T	45	10	81	Right	+13
13	1 & 2	3	V, T	11	0	23	Right	−13
14	1 & 2	4	V	98	97	100	Right	−3.5
15	1 & 2	37	V, T	17	0	35	Right	+12.40
16	1 & 2	12	V	23	0	46	Right	+49.4^†^
17	1	NA	T	88^†^	90^†^	86^†^	Right	−8.5
18	1 & 2	39	V, T	2	0	4	Right	+7.58
19	1 & 2	1	V	39	10	69	Right	+2.66
20	1 & 2	1	0	16	0	31	Right	+2.41
21	1 & 2	1	0	80	87	73	Right	+2.33
22	1	2	0	4	0	8	Right	+0.5
23	2	10	V	70	63	78	Right	+8.6
24	2	1	V, T	55	13	96	Right	+5.33
25	2	1	V	0	0	0	Right	+24.5
26	2	12	T	0	0	0	Right	NA

Screening performed on the ward in acute stage or within 3 months from stroke.

Star cancellation = proportion of identified targets on the left- and right-hand side of the array on the star cancellation task.

NA = not available.

V = visual.

T = tactile.

* = Score on the Mesulam Shape Cancellation.

† = Test performed in the chronic stage.

### 2.1 Behavioural study

Right-hemispheric stroke patients took part in the behavioural study (Table 2, IDs 1–21). Their neurological presentation has been summarised in Supplementary material 6.

**Table 2:** **Demographic and clinical data for patients who took part in Study 1 (Behavioural study) and Study 2 (Imaging study).** A total of 117 right-hemispheric stroke patients were screened at Charing Cross Hospital in London between September 2015 and August 2019. All patients who took part in the studies had normal or corrected-to-normal vision, with no history of neurological or psychiatric diseases. 18 patients participated in both studies. Study 1: mean age 65.55 ± 11.72 years old; age range 44 – 85 years old; F = 10; 20 right-handed. Study 2 (patients): mean age = 65.77 ± 12.37 years old; age range 45–85 years old; F = 11. Study 2 (healthy controls): mean age = 63.00 ± 8.14 years old; age range 49 – 80 years old; F = 10. Sex: F = female, M = male. Hand: R = right-handed L = left-handed. M (UL) = motor upper limb. Six patients had upper limb hemiparesis and 4 were using wheelchairs at the time of participation.

ID	Study	Age	Sex	Hand	Aetiology	Lesion location	Months post-stroke
1	1 & 2	77	M	R	I	F-T-P	48
2	1 & 2	69	F	L	H	Th, BG	30
3	1 & 2	77	F	R	I	F-T-P, BG	10
4	1 & 2	71	M	R	I	IC	27
5	1	61	M	R	H	T	3
6	1 & 2	57	F	R	I	T-O, h, Th, CC	6
7	1	72	M	R	I	T-P	3
8	1 & 2	54	F	R	I	CR, LN	5
9	1 & 2	85	F	R	I	BG, insula, fO	36
10	1 & 2	63	M	R	I	T-P, insula, BG, fO	5
11	1 & 2	46	F	L	I	F-T-P	48
12	1 & 2	44	M	R	H	BG, CR	23
13	1 & 2	76	F	R	I	F-T-P, CS	3
14	1 & 2	54	M	R	H	T	5
15	1 & 2	75	F	R	I	F-T, IC, LN	26
16	1 & 2	85	M	R	I	P-O	3
17	1	74	F	R	I	F-T-P, BG, insula	11
18	1 & 2	65	M	R	H	Th, IC	19
19	1 & 2	59	M	R	I	T-P, insula	3
20	1 & 2	55	M	R	H	Th	6
21	1 & 2	54	M	R	I	F, CR, insula, fO	3
22	1	69	F	R	H	BG	4
23	2	77	F	R	I	P-O	84
24	2	80	F	R	I	BG, IC	3
25	2	70	F	R	I	F-T-P, insula, fO, tO	3
26	2	53	M	L	I	F-P, insula	3

**Fig. 2:**
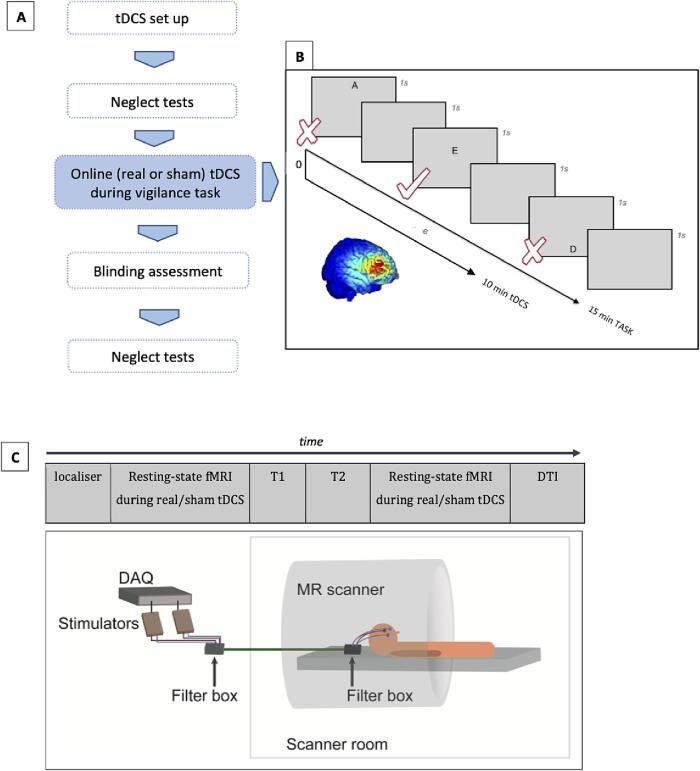
Experimental procedures. A. Experimental set-up for the behavioural study. The vigilant attention task was programmed in E-prime 2.0 (Psychology Software, Inc.) and administered on a 14-inch screen laptop (HP EliteBook 8460p) at a viewing distance of ∼56 cm. Participants were presented with a sequence of black letters (A-E, each ∼15 × 15 mm) displayed sequentially on a uniform grey background (Fig. 2B). The paradigm comprised 450 trials in total: 180 targets and 270 distractors. Button presses and reaction times (RT) were recorded. Before starting the task, patients completed a practice run until accuracy was at 100 % for 10 consecutive trials. In a double-blind sham-controlled crossover design, patients performed the vigilant attention task (15 min) whilst receiving 10 min of real/sham tDCS, in counterbalanced order. That is, if they received real tDCS in the first session, they would receive sham in the second (on a separate day, range 6–104 days apart) and vice versa. Stimulation order was randomised using a random order matrix set by a researcher who did not deliver tDCS. Stimulation was initiated by running a script specifying the condition (real or sham) for each participant. To maximise blinding, patients were gently habituated to the stimulation procedure at the beginning of each session: tDCS was delivered for 10 s at half intensity (0.5 mA) followed by 10 s at full intensity (1 mA). Real tDCS was delivered using 1.5 cm disc electrodes connected to a neuroConn DC stimulator with a ramp of 10 s up to 1 mA, full intensity at 1 mA for 10 min and a ramp down over 0.5 s. Sham tDCS consisted of the ramp up and ramp down stages only. Caffeine consumption 4 h before participation was not allowed. B. Sequential test display for the behavioural paradigm. Letters were displayed at one of five possible locations along the vertical meridian of the screen and patients had to make a button press to stimuli appearing at target locations, regardless of letter identity, by pressing the central button on a response box when a letter was displayed at one of two target locations on the screen. They were instructed to withhold responses to letters presented at the three non-target locations. The first test display shows a letter appearing at one of the non-target locations; the third shows a letter at a target location; the fifth shows a letter at a non-target location. Presentation order was randomised as follows: 5 different letters were displayed in one of the 5 possible locations 90 times, in random order. Letters were displayed at a fixed rate every 2 s, remaining on the screen for 1 s. C. Experimental set-up for the imaging study. MRI acquisition timeline and schematic representation of the procedure, showing that real and sham tDCS were administered during two resting-state functional MRI runs (eyes closed), in counterbalanced order over the course of 1 fMRI session on a single day. TDCS was set up on participants’ head prior to entering the scanner room and kept in position with conductive paste and padding around the head.

A randomised, double-blind, crossover design study was employed, consisting of two separate counterbalanced sessions (real/sham tDCS at 1 mA) ≥ 7 days apart (mean 24 days, range 7–104) (Fig. 2). TDCS was delivered during the first 10 min of a 15-minute attentional task. This allowed tracking of any tDCS effects during and following cessation of stimulation. The task was adapted from a non-lateralised go/no-go computer task (Malhotra, Coulthard, & Husain, 2009) previously shown to detect non-lateralised attentional deficits and the *vigilance decrement*, a decline in performance with increasing time-on-task, in right hemisphere patients (Mackworth, 1948, Malhotra et al., 2009, Parasuraman et al., 1998). The stimulation montage consisted of three circular electrodes (15 mm Ø) with the anode over F4 and return electrodes over F8 and FP2 (10–20 EEG International system) (Supplementary material 2).

Although our primary outcome was the computerised attentional task indexing non-lateralised vigilant attention, we also carried out a short cognitive battery consisting of three standard clinical measures of lateralised deficits (star and symbol cancellation, line bisection task) before and after tDCS (Fig. 2A). Integrity of blinding and side effects were assessed using a validated questionnaire (Brunoni et al., 2011).

#### 2.1.1 Behavioural data analysis

Statistical analyses of behavioural data were conducted using SPSS Statistics v26.0 (IBM, 2019). Data distribution was assessed using the Kolmogorov-Smirnov test; homogeneity of variance was assessed using Levene’s test. Anticipatory responses (RT < 200 ms) were removed. When the assumption of normality was violated, scores were log-transformed. A series of repeated measures analysis of variance (ANOVA) with Order (real-sham vs sham-real) as between-subjects factor and Condition (real vs sham tDCS) as within-subjects factor was performed for omissions (i.e., missed targets), commission errors (i.e., false alarms), mean RT, RT variability (RTvar = RTstandev/RTmean) and accuracy (proportion of correct responses). We also calculated the following measures from signal detection theory: target sensitivity (*d’*=z(hits)-z(FA)) and response bias or criterion (C = z(hits) + z(FA)/2). One key feature of vigilant attention, in healthy individuals and patient groups, is the *vigilance decrement*, a drop in performance with increasing time on task. To examine this directly, task duration was divided into three equal epochs of approximately 5 min and ANOVAs were carried out including Epoch (1 vs 2 vs 3) as within-subjects factor. Effect sizes for significant effects (p <.05) were calculated using partial eta squared (η_p_^2^). Bonferroni-corrected post-hoc tests were used to examine significant effects. Paired t-tests were used to compare response bias during real and sham tDCS.

To analyse clinical measures of spatial bias, assumption-free statistical tests were used due to skewed data distribution that persisted after logarithmic transformation. To evaluate the effect of tDCS on continuous and categorical variables, Friedman’s ANOVA and Pearson’s chi-square tests were used.

Perception of stimulation was analysed via a series of Pearson’s chi-square tests of independence. The intensity of each reported side effect was analysed via a repeated measures ANOVA with Stimulation (real vs sham tDCS) as within-subjects factor.

### 2.2 Imaging study

22 right-hemispheric stroke patients and 22 age-matched healthy controls took part in the imaging experiment (Table 2). Structural and functional brain scans were acquired in a single session (Fig. 2C). High-resolution T1- and T2-weighted, T2*-weighted gradient-echo echoplanar imaging and diffusion tensor imaging (DTI) sequences were acquired on a 3 T Siemens Verio using a 32-channel head coil. Two runs of functional MRI were acquired with participants receiving real and sham tDCS (counterbalanced order) while at rest with eyes closed, having been instructed to stay still without falling asleep. Probing of tDCS blinding efficacy was carried out via intercom using a validated questionnaire after each fMRI run.

#### 2.2.1 Imaging data analyses

All fMRI data were preprocessed and analysed using FSL Version 5.0.10 from the FMRIB Software Library (Smith et al., 2004) (Supplementary material 4). We created univariate models to examine the influence of different factors (lesion volume and location, functional connectivity during real/sham tDCS) on task performance and response to tDCS in the Behavioural study. For anatomical correlates of task performance, we corrected resulting maps using permutation-based thresholding set to 5000 (Winkler, Ridgway, Webster, Smith, & Nichols, 2014). Tests were performed one-tailed. Data were segmented into 150 regions-of-interest using the AALCAT atlas (Catani and Thiebaut de Schotten, 2008, Tzourio-Mazoyer et al., 2002).

## 3 Results

### 3.1 Behavioural study

#### 3.1.1 Errors

On average, patients made 86 errors (out of 450 trials) throughout the task. 60 % errors were omissions and 40 % were false alarms. They were less accurate [F(1,42) = 22.456, p <.01] and slower [F(1,42) = 25.544, p <.01] than a control group of 21 age-matched healthy controls (mean age 66.24 ± 9.30 years old; age range 51–81 years old; F = 8).

**Fig. 3:**
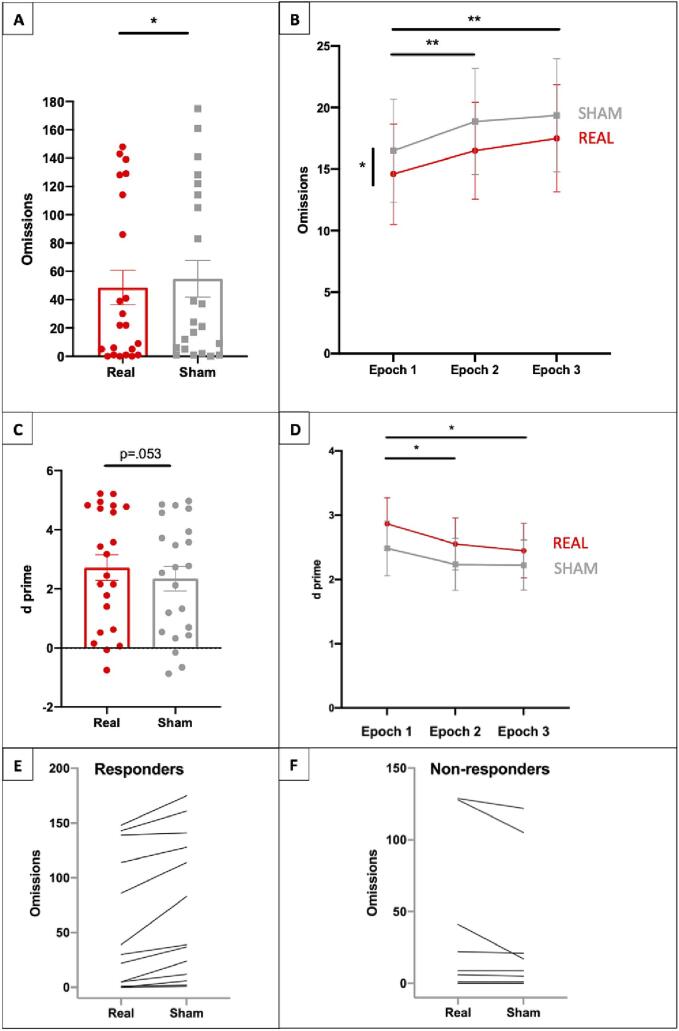
Tdcs effects and the vigilance decrement. A. Total number of task omissions made by patients, at group level, during real and sham stimulation. B. Omission rate across epochs. Each epoch was approximately 5 min duration. There were fewer omissions throughout the duration of the task in the real stimulation session but performance still worsened with time on task, indicating that the vigilance decrement was not abolished. C. Target sensitivity (d’) exhibited by patients during real and sham stimulation. D. Target sensitivity across epochs. A vigilance decrement was observed in both sessions. E. Task omissions for responders (n = 13); F. Task omissions for non-responders (n = 9). Asterisks indicate statistically significant differences.

ANOVA of patients’ omissions showed a significant main effect of Stimulation [F(1,20) = 4.608, p =.044, ηp2 = 0.187], with fewer omissions during real (mean 48.95, SEM 12.17) versus sham tDCS (mean 54.73, SEM 12.19) (Fig. 3A). There was no significant effect of Order [F(1,20) = 4.123, p =.056] or Stimulation x Order interaction [F(1,20) = 0.356, p =.558]. ANOVA of false alarms did not reveal any significant main effects or interactions [all p values > 0.05]. ANOVA of target sensitivity (d') revealed that the main effect of Stimulation was close to significance [F(1,20) = 4.229, p =.053, ηp2 = 0.175]: patients performed at a *d’* of 2.716 (SEM 0.430) during real tDCS and of 2.348 (SEM 0.414) during sham tDCS (Fig. 3C). For *d’*, the main effect of Order was significant [F(1,20) = 5.586, p =.028, ηp2 = 0.218], with patients in the real-then-sham group performing at higher sensitivity levels (mean 3.502, SEM 0.330) as compared to those who received treatment in the opposite order (mean 1.724, SEM 0.404). The interaction between Stimulation x Order was not significant [F(1,20) = 1.740, p =.202]. A paired *t*-test examining response criterion during real and sham tDCS revealed no significant effects of Stimulation [t(21) = -0.805, p =.430], indicating no difference in response bias between stimulation conditions.

#### 3.1.2 Reaction time

ANOVA revealed a main effect of Order [F(1,20) = 7.294, p =.014, ηp2 = 0.267] on RT: patients in the group that received sham-then-real tDCS were slower to respond (mean 804.415, SEM 37.324), as compared to those who received real-then-sham tDCS (mean 627.632, SEM 23.841). There was no main effect of Stimulation [F(1,20) = 0.082, p =.778] or interaction between Stimulation x Order [F(1,20) = 2.168, p =.156] for RT. ANOVA conducted on RT variability did not reveal any significant effects [all p values > 0.05].

#### 3.1.3 Time-on-task

Task duration was divided into three time-epochs of approximately 5 min. ANOVA revealed a main effect of Epoch for omissions [F(2,42) = 9.873p =.000, ηp2 = 0.320] and *d*’ [F(2,42) = 7.500, p =.002, ηp2 = 0.263], indicating that patients showed a vigilance decrement (Fig. 3B and 3D). Performance was worse in Epoch 2 versus Epoch 1 (p <.05) and in Epoch 3 versus Epoch 1 (p <.05) but not Epoch 2 (p >.05). The interaction between Stimulation x Epoch was not statistically significant, suggesting that stimulation did not abolish the decrement but affected performance throughout task duration.

#### 3.1.4 Clinical tests

Patients performed clinical tests before and after real/sham tDCS (i.e., offline). Total score on cancellation tasks did not significantly differ across the four testing sessions [Star: χ2(3) = 4.241, p =.237; Mesulam: χ2(3) = 6.369, p =.095]. The number of targets found on the left vs on the right side of the arrays was also not significantly different across sessions [Star: χ2(3) = 3.082, p =.379, with CoC χ2(3) = 1.255, p =.740; Mesulam: χ2(3) = 3.162, p =.367]. Friedman’s two-way analysis of variance by ranks was used to compare displacement on the line bisection task. No significant differences were observed after Bonferroni correction [all p values > 0.05].

#### 3.1.5 Lesion anatomy

Lesion volume extracted from lesion maps delineated in native space did not correlate with omission rates [r(22) = 2.11, p =.346] or tDCS response, as measured by the difference in omissions between real and sham tDCS [r(22) = 0.312, p =.157]. Univariate statistical comparison showed that damage to the right cingulum and corpus callosum was associated with the number of task omissions (respectively, z = 3.30 and z = 3.44).

**Fig. 4:**
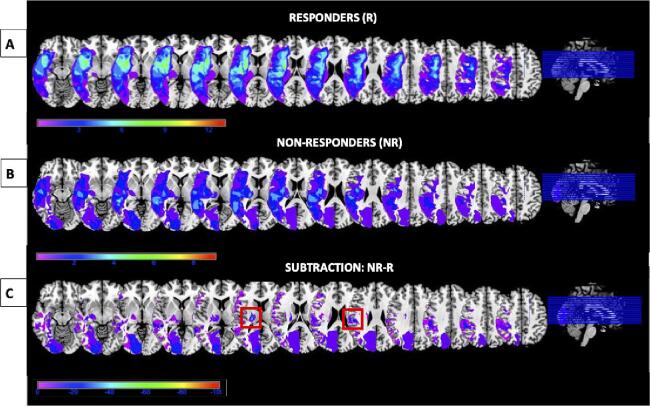
Neural correlates of tDCS response. Anatomical images (high-definition MPRAGE for 18 patients, clinical T1 MRI scans for 4 patients) were used for lesion analyses. Lesion maps were delineated on native space using the ImSeg Interactive Image Segmentation Tool (v1.8) developed by Dr Ben Glocker (Imperial College London). Binary lesion maps were normalised to a common stereotaxic space using the spatial normalisation routines of the Clinical Toolbox in SPM. Standard normalisation procedure and unified segmentation-normalization with enantiomorphic normalisation were employed for low-resolution images and high-quality images respectively. A. Lesion overlap for patients who performed better with real versus sham targeted tDCS (responders, n = 13). B. Lesion overlap for patients who performed worse or the same with real versus sham targeted tDCS (non-responders, n = 9). C. Lesion subtraction map showing the percentage of lesion overlap of non-responders after the subtraction of the overlap of responders. In light blue (inside the red squares), the right thalamus and the right postcentral gyrus which were associated with a lack of benefit from tDCS (MNI coordinates: X = 20, Y = -20, Z = 12 and X = 55, Y = –22, Z = 28 respectively). All resulting plots have been overlaid onto an MNI template brain. Colour bars reflect the relative frequency of damage. Note that although the frontal lobe may have been partially lesioned in patients, the area underneath the electrodes was spared as confirmed by a Consultant Neurologist who examined clinical scans prior to patient enrolment.

By subtracting total number of task omissions during real tDCS from the number of omissions during sham tDCS, we classified patients into tDCS Responders (R(n = 13)) and Non-Responders (NR(n = 9)) (Fig. 4). Lesion volume was not significantly different between the R and NR groups (mean volumes 65 cm^3^ and 45 cm^3^ respectively) [F(1,21 = 0.797, p =.383)]. To identify regions associated with lack of response, we performed an exploratory lesion subtraction (Lee et al., 2019). The two areas associated with lack of response to tDCS (i.e., most frequently damaged in the NR group and spared in the R group) were the right thalamus and the right postcentral gyrus (Fig. 4C).

#### 3.1.6 tDCS blinding

Perception of stimulation was at chance for patients [χ2(1) = 0.419, p =.517]. General discomfort was very low, and the intensity of all side effects was on average absent or mild [always below 1, on a scale from 0 to 4].

### 3.2 Imaging study

#### 3.2.1 Whole-brain connectivity

Functional correlates of tDCS were examined by comparing whole-brain functional connectivity during real and sham tDCS applied at rest in the MRI scanner.

Using a data-driven parcellation, voxels were separated into a set of nodes at a dimensionality of 30 components, and 14 large-scale resting state networks which were common across participants were identified via cross-correlation with an atlas (Shirer, Ryali, Rykhlevskaia, Menon, & Greicius, 2011). 5 large-scale networks of interest were selected for further analysis: salience network (SN), involved in detection and selection of salient stimuli; left and right executive control network (LECN and RECN), implicated in goal-oriented behaviour and cognitive control; visuospatial network (VSN), engaged in orienting to salient visuo-spatial information; and default mode network (DMN), with a role in introspection and mind wandering. These specific networks were selected because they included the stimulation site or its homologue, or because they had a spatial distribution associated with the attention function.

The dual regression approach was then used to identify subject-specific and condition-specific contributions to group-level Independent Component Analysis (ICA). We directly compared whole-brain functional connectivity (FC) spatial maps: by testing for shape and amplitude of low frequency fluctuations, this approach provided a voxel-wise measure of FC that reflected the correlation between each voxel and the rest of the network. Contrast matrices were created using the general linear model framework: the dependent variable contained all values from a single voxel across subjects’ spatial maps. ICA was performed separately for each group: 17 stroke patients and 19 healthy older adults.

Permutation-based thresholding was used to correct result maps using a large number of permutations (Mirman et al., 2018). This correction has been recommended by leading experts in neuroimaging, as it offers the same control as the Bonferroni correction when performing a huge number of statistical tests but has better statistical power when individual voxels are not truly independent (Karnath, Sperber, Wiesen, & de Haan, 2019). Comparisons were run using FSL randomise nonparametric permutation testing (Winkler et al., 2014), with 5000 permutations and a threshold-free cluster enhancement method to control for multiple comparisons. Corrected p-values were fully corrected for multiple comparisons across voxels for each network.

**Fig. 5:**
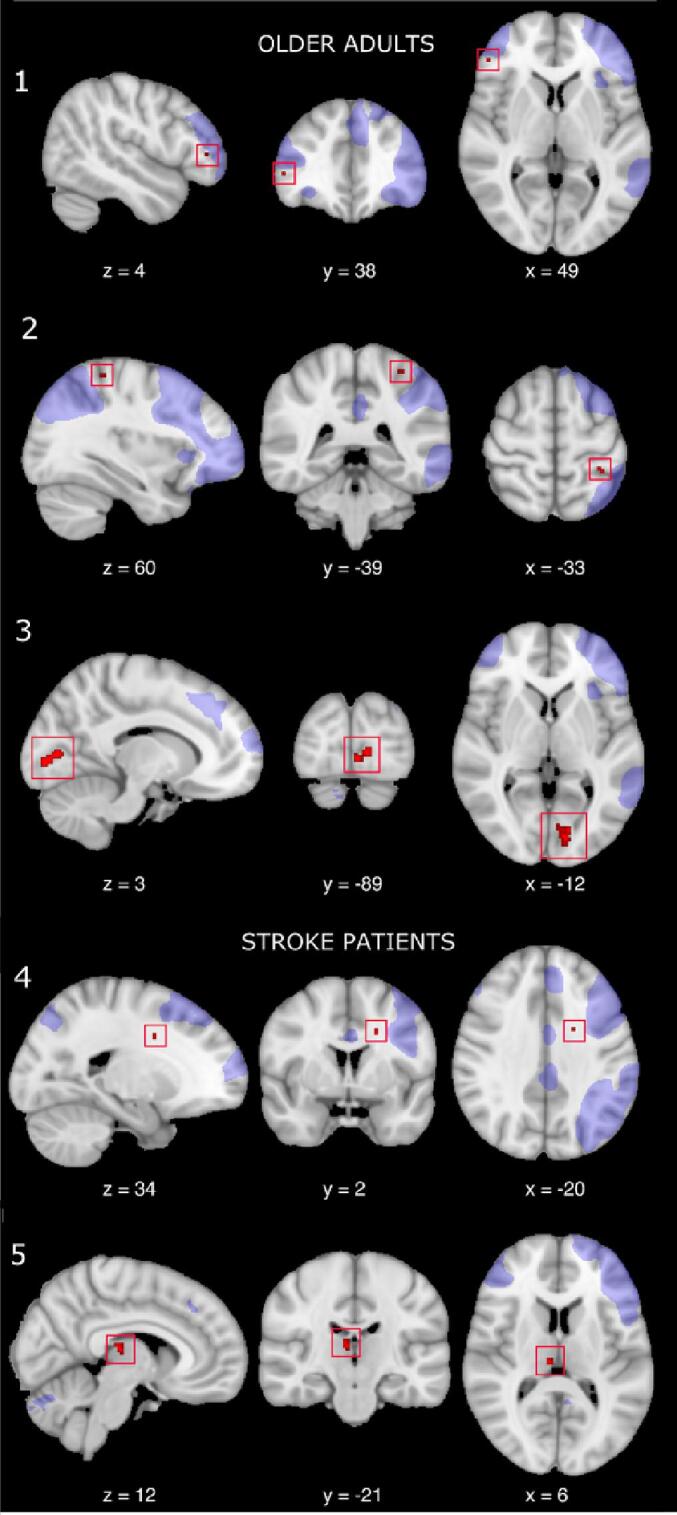
Increased connectivity within the left executive control network during tDCS. Results from the whole-brain analysis showing larger functional connectivity within the left executive control network (in shaded purple) during real as compared to sham tDCS in healthy age-matched controls, with clusters found in right inferior frontal gyrus (1), left posterior central gyrus (2) and left occipital lobe (3), and stroke patients, with clusters found in left superior corona radiata (4) and right thalamus (5). X, Y and Z refer to MNI coordinates for peak of difference in connectivity. All resulting plots have been overlaid onto a 1 mm MNI brain.

A series of one-sample t-tests was performed on the set of 4D spatial volumes to assess for connectivity differences between real and sham tDCS. Difference maps were found to be significantly different from zero in the LECN across both groups [older adults: p =.009; stroke patients: p =.026]. Clusters of higher connectivity within LECN were detected during real versus sham tDCS in different regional patterns for patients and age-matched healthy controls (Fig. 5). For patients, these were in the right thalamus and left corona radiata; for controls, in right prefrontal and left parieto-occipital cortices. In the healthy group, further increases in functional connectivity were also found during real versus sham tDCS within the SN (p =.034) and VSN (p =.008). Specifically, within the SN, a cluster of larger connectivity was found in the left mid cingulum; within the VSN, clusters were identified in the right supplementary motor area, in the left superior frontal gyrus and postcentral gyrus. There was no significant difference in FC response to tDCS for any of the five networks of interest between healthy controls and stroke patients [all p values > 0.05]. A control analysis carried out in healthy controls to exclude contamination of brain networks established that the two sham runs were comparable, regardless of stimulation order (Supplementary material 5).

#### 3.2.2 Average network connectivity

**Fig. 6:**
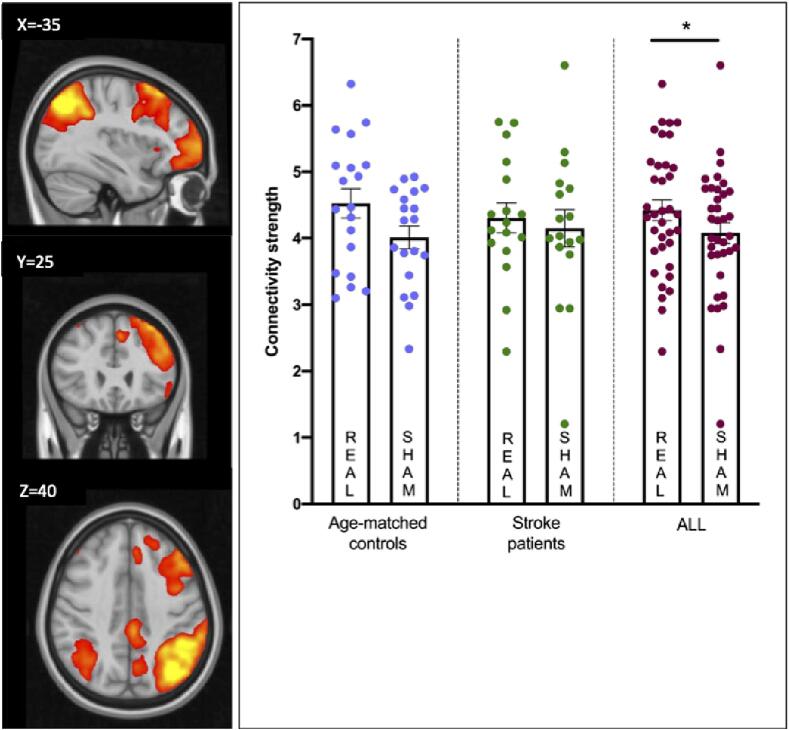
Intrinsic mean functional connectivity strength within the LECN during real and sham tDCS for stroke patients and healthy age-matched adults. Mean FC for the Left Executive Control Network (LECN, in scale yellow to red) for the patient group (left panel). Results have been overlaid onto a 1 mm MNI brain. The main effect of stimulation is shown, with larger connectivity observed during real tDCS as compared to sham tDCS across all groups (maroon dots) (right panel). Individual datapoints are also shown for age-matched healthy controls (lavender dots) and stroke patients (green dots) separately. Bars represent mean and SEM.

Next we assessed intrinsic mean connectivity strength within brain networks. The BOLD time series for voxels within each network was extracted. We masked the output z-maps of dual regression, which reflect subject- and condition-specific strength of FC for each network, by the group average maps of the respective component. Connectivity strength during real and sham tDCS was contrasted via a series of repeated measures ANOVA with Group (patients vs healthy) and Stimulation (real vs sham) as factors. A main effect of Stimulation emerged for the LECN [F(1,34) = 7.674, p <.01, ηp2 = 0.184], with higher connectivity observed during real (mean + 4.4) versus sham tDCS (mean + 4.1) (Fig. 6). The main effect of Group and the interaction between Group x Stimulation were not significant [p >.05]. For all other networks of interest, mean connectivity strength was not significantly different between real and sham tDCS. These findings were replicated using independent masks derived from an anatomical atlas (Shirer et al., 2011).

#### 3.2.3 Group differences

For between-groups differences, likely to be associated with the presence of a brain lesion rather than an effect of tDCS, main effects of Group for the SN (F(1,34) = 16.53, p <.01, ηp2 = 0.322) and the DMN (F(1,34) = 5.894, p <.05, ηp2 = 0.148) emerged, with larger connectivity strength in healthy adults (respectively, mean + 3.647, +4.398) versus stroke patients (respectively, mean + 2.873, +3.816).

#### 3.2.4 Local connectivity changes

**Fig. 7:**
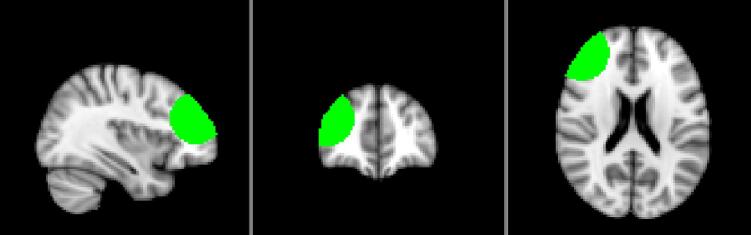
MNI brain template with a mask superimposed (in green). The mask is centred around the stimulation target (the right dorsolateral prefrontal cortex). It was created using the software MANGO (Multi-image Analysis GUI) by defining a 25 mm radius sphere that triangulated the EEG electrodes. Coordinates for the electrodes were obtained from their projection onto the cortical surface and converted to MNI space using the Nonlinear Yale MNI to Talairach Conversion Algorithm (F4: x  = 42, y = 24, z = 46; F8: x  = 55, y = 30, z = -1; FP2: x  = 24, y = 66, z = 12). Areas outside the brain were removed. (For interpretation of the references to colour in this figure legend, the reader is referred to the web version of this article.)

Mean FC strength in the region underneath the electrodes was extracted using a mask encompassing the electrode contact regions (Fig. 7). Networks of interest showing overlap with this mask were the SN, the DMN and the RECN. ANOVA of connectivity strength during real and sham tDCS for the masked region showed no significant effects for any networks in patients and healthy controls [all p values > 0.05].

#### 3.2.5 Behaviour:connectivity relationship

Finally, we explored the relationship between behavioural (indexed as task accuracy) performance and functional connectivity changes during tDCS in 14 patients who took part in both studies and met censoring criteria for movement. Mean differences in functional connectivity were extracted for each network using an anatomical mask (Shirer et al., 2011). Network strength was correlated to difference in task accuracy. To correct for multiple comparisons, permutation thresholding was used to control for the family-wise error rate and set to 5000 (Winkler et al., 2014). All statistical tests performed were one-tailed, under the assumption that brain injury would not have led to improved test performance. Changes in performance were strongly correlated with diminished activation within the DMN [r = -0.452, p (two-tailed) = 0.006] and an increase in connectivity within the RECN [r=+0.496, p(two-tailed) = 0.002].

#### 3.2.6 tDCS blinding

Perception of stimulation delivered in the MRI scanner was at chance for stroke patients [χ2(1) = 0.14, p =.705] and age-matched healthy participants [χ2(1) = 2.78, p =.095].

## 4 Discussion

We examined behavioural and brain network response to high-definition electrical stimulation of spared cortex in a group of right-hemispheric stroke patients with neglect. Previous research has shown that this population suffers from persisting attentional deficits that correlate with reduced network connectivity (Barrett, Boukrina, & Saleh, 2019). Deficits are frequently non-lateralised, persisting and linked to reduced post-stroke recovery, and could be targeted by neurorehabilitation approaches (Mole and Demeyere, 2020, Nurmi et al., 2018). On this basis, we pragmatically recruited patients with neglect (Nurmi et al., 2018) and evaluated whether non-lateralised attentional performance could be modulated by right prefrontal tDCS, following on from previous studies in healthy populations (Brosnan et al., 2018, Nelson et al., 2014).

We employed a tDCS montage that would maximise targeting of spared regions, minimising current dissipation. In our opinion, this targeted approach is particularly important in the context of carrying out stimulation in patients with brain lesions, although we note that it is impossible at present to perfectly estimate current spread in this patient population. By targeting intact cortex involved in the ability to maintain attention, we aimed to influence activity in large-scale attention networks that are typically damaged in neglect.

Using a double-blind sham-controlled crossover study and a concurrent tDCS-fMRI study, we found that precise targeting of spared prefrontal cortex improved target detection on an attentional task and that stimulation increased functional connectivity within large-scale networks.

### 4.1 tDCS reduces task omissions

In a group of stroke patients with neglect, tDCS targeted to the right DLPFC improved target detection on a non-lateralised attentional task. The effect outlasted stimulation duration for at least 5 min, with superiority of real stimulation as compared to sham stimulation.

One possible explanation for the effect of tDCS on error rate is that it acted via increasing general alerting and arousal levels on a trial-by-trial basis, similar to the effect of behavioural interventions for neglect (Olgiati et al., 2016, Robertson et al., 1998). As noted in Malhotra et al (2009), inhibitory control is involved in accurate performance on this task. However, the effect of tDCS was to reduce omissions rather than false alarms, suggesting that stimulation did not improve inhibitory control. No change in RT was observed during tDCS, as might be expected if the effect was arousal-mediated (Luna, Román-Caballero, Barttfeld, Lupiáñez, & Martín-Arévalo, 2020). Concurrent physiological measures, such as pupillometry, could help clarify this in future work. It is also possible that the effect of tDCS on omissions was mediated by modulation of the interplay between networks involved in non-spatial and spatial attentional processes, which is necessary for performance on this task (Corbetta & Shulman, 2011). The tDCS montage, targeting the right DLPFC, may have facilitated fronto-parietal coupling, influencing activity in posterior areas (Cazzoli et al., 2020).

### 4.2 Vigilance decrement is not susceptible to tDCS

A comparison of task performance between patients and an age-matched control group was run to check that the task was able to elicit a vigilance decrement (i.e., a decline in performance with decreasing time-on-task) in patients, as in Malhotra et al., 2009. As expected, patients were slower and less accurate than controls, and their performance was characterised by a vigilance decrement. Data from a parallel study with healthy volunteers is included in a separate manuscript that is being prepared for submission. In that dataset, false alarms represented the vast majority of errors.

As expected, right hemisphere stroke patients showed a *vigilance decrement* on this task, with attention declining with time-on-task. This decrement was not modulated by the application of brain stimulation. This finding contrasts with those from previous work in younger healthy volunteers (Luna et al., 2020, Nelson et al., 2014) but is in keeping with research with older adults showing that tDCS modulates attention throughout a task without abolishing the decrement (Brosnan et al., 2018).

### 4.3 Both groups responded to treatment

The analysis also revealed a main effect of Order, with patients receiving the treatment in the sham-then-real order responding more slowly and less accurately than the group who received the same treatment but in reverse order. Although patients were randomly assigned to a treatment order (Real-then-Sham or Sham-then-Real), it is still possible that the two groups were not perfectly matched in terms of attentional performance.

A baseline session would have been helpful to balance patient assignment to treatment arms. We did not include such a baseline session as it would have increased the potential effects of task practice and, more importantly, increased demands on patients and dropout rate. It is however critical to highlight that the effect of Order group was never found to interact with that of stimulation in any outcome measure. This suggests that stimulation had an effect on performance in both groups.

### 4.4 tDCS modulates contralesional functional connectivity

In a parallel imaging study, we examined BOLD correlates of tDCS in patients and age-matched healthy adults using a completely data-driven approach. We found that the network-modulatory effects of targeted tDCS were not local: functional connectivity for the area underneath the electrodes was unaffected by tDCS. Instead, stimulation modulated large-scale distributed brain networks, influencing connectivity in the contralesional hemisphere, specifically within the LECN. This result was arrived at using two independent approaches, the whole-brain FC and intrinsic network strength analyses. Within the LECN, different clusters of larger mean connectivity were identified in both hemispheres during real versus sham stimulation.

This is the first work to show online modulation of a contralateral network by HD-tDCS in a population of brain-injured individuals and in age-matched healthy adults. These findings are consistent with previous work showing that anodal stimulation of the right DLPFC causes bilateral modulation of thalamocortical networks (Keeser et al., 2011, Sankarasubramanian et al., 2017), and are in keeping with our exploratory lesion mapping, which showed the importance of thalamic integrity for tDCS response. Our results extend the emerging body of work indicating a widespread, large-scale network-level impact of tDCS, extending from the stimulation site to remote but connected areas (Cabral-Calderin et al., 2016, Li et al., 2019).

### 4.5 Network correlates of behavioural response

Analysis of treatment response showed that the degree of tDCS-induced decreased connectivity within the DMN and larger connectivity within the RECN were linked with greater behavioural response to tDCS. The DMN data are of particular interest as previous work in traumatic brain injury patients showed a link between vigilance lapses and DMN activation, especially within the precuneus and posterior cingulate cortex, strongly connected to the prefrontal cortex via the cingulum (Bonnelle et al., 2011). Our findings, showing vigilance deficits associated with cingulum damage and tDCS-induced vigilance improvement related to DMN deactivation, are in keeping with this work.

It is important to note that tDCS was applied during a task in the behavioural study and at rest in the scanner. Although the mechanisms underlying the interaction between tDCS and task-induced activity are not clear (Fehring et al., 2019), it is likely that there will be some difference in the neural correlates of the two situations because of this difference.

### 4.6 Neural correlates

An important question in the tDCS literature relates to the inter-individual differences in response to stimulation. At the individual level, patient trajectories in response to tDCS varied. Full understanding of individual characteristics influencing response to treatment is of particular importance in patient studies, particularly in heterogenous syndromes such as neglect. Performance on the vigilance task was found to depend on lesion location, rather than general lesion volume. Two large white matter tracts, the right corpus callosum and the right cingulum, emerged as key, suggesting a potential role of inter-hemispheric and fronto-parietal disconnections in vigilant attention dysfunction. Lesion anatomy also showed that patients who did not respond to tDCS had lesions in the right thalamus and postcentral gyrus, where damage was linked to reduced tDCS response. The thalamus is considered critical for response inhibition and reward processing, which modulate attention (Graybiel, Aosaki, Flaherty, & Kimura, 1994). Portas and co-workers found increased attention-related activity in the thalamus in conditions of low arousal, postulating a role for the thalamus in mediating the interaction between arousal and attention (Portas et al., 1998). Focal thalamic lesions have been found to cause neglect (Karnath, Himmelbach, & Rorden, 2002). A role for the thalamus is also supported by our network analysis: during real tDCS, the right thalamus showed an increase in connectivity to regions within the LECN. We also found that the right postcentral gyrus, incorporating primary somatosensory cortex, was involved in tDCS response. As proprioceptive gaze input has been revealed for this gyrus, there is a possible role of this area in spatial attention (Wang, Zhang, Cohen, & Goldberg, 2007). Considering that the task used in this study has a spatial, albeit non-lateralised, component, tDCS may have exerted its effect by boosting this aspect of task performance.

### 4.7 Study limitations

Our study has a number of limitations. Although it has been proposed that tDCS may foster brain circuitry reorganisation after a stroke (Vallar & Bolognini, 2011), its use remains controversial (Horvath et al., 2015, Learmonth et al., 2021, Opitz et al., 2017) and its clinical effectiveness is debated (Cappon et al., 2016, Kekic et al., 2016). As the study was designed to examine the effects of stimulation on non-lateralised attention, we did not examine the effect of tDCS on lateralised attention *during* stimulation. Thus, it remains unknown whether tDCS can modulate performance on a standard clinical task for neglect if clinical tests are administered during stimulation. Also, this study aimed at evaluating the effect of a single treatment application; future studies need to establish whether consecutive applications of tDCS produce a carryover effect that translates to meaningful clinical improvement. A limitation stems from the decision to split patients into responders and non-responders on the basis of a change in performance. Different outcomes would be yielded dependent on the cut-off points used to categorise a response. However, considering patient heterogeneity, it is unlikely that a single treatment will work for every patient, and it would be clinically helpful if future studies would be able to predict who will benefit from the application of each set of parameters of brain stimulation (responders), and who will not. A further limitation concerns the interpretation of the behavioural changes during tDCS and network changes during tDCS. For pragmatic reasons, we used resting state fMRI in stroke patients across the disability spectrum to minimise patient burden and maximise participation. This design does not allow us to directly link findings from the two studies. More research in the direct comparability of the effects of online and offline tDCS are needed (Olgiati & Malhotra, 2020).

Results from this proof of principle study are encouraging, and replications with larger sample sizes would now be important to confirm our neuroimaging findings. Considering the well-known heterogeneity of this patient group, and the likelihood that they will respond differently to treatment, the cohort is anticipated to be large. A possible extension could be to stratify patients into groups with similar functional/structural connectivity, and explore treatment effects in each subgroup (e.g., see Li et al., 2019).

### 4.8 tDCS for the treatment of neglect

Despite the above mentioned limitations, our multimodal study adds to the emerging body of literature on prefrontal HD-tDCS being able to modulate non-lateralised attention (Luna et al., 2020). Although there is not yet robust evidence that tDCS is efficacious in neglect, preliminary evidence suggests that it can improve attentional deficits when applied as a stand-alone technique and in synergistic application with other therapies (for recent reviews, see Olgiati and Malhotra, 2020, Zebhauser et al., 2019). In the current work, patients could not distinguish stimulation from the sham condition, demonstrating that blinding was successful. Given that this clinical population often has large lesions, we modelled a HD-tDCS montage that allowed precise targeting of spared regions, minimising current dissipation. Importantly, we were able to safely test severely affected individuals, suggesting that the findings generalise to the wider population of stroke survivors. In this scenario, a high quality randomised clinical trial is justified to compare a range of treatment healthcare interventions (including tDCS combined with a behavioural intervention) for attentional impairments following right-hemispheric stroke.

### 4.9 Conclusions

We aimed to translate recent advances in tDCS research to test a clinical population typically affected by significant attentional deficits. Our results show that a HD-tDCS intervention reduces attentional deficits following right hemisphere stroke. Integration of tDCS with concurrent neuroimaging allowed us to identify the brain connectivity changes induced by stimulation. Further work is warranted to determine whether repeated stimulation can bring about longer lasting effects with associated improvement in daily function.

### CRediT authorship contribution statement

**Elena Olgiati:** Conceptualization, Data curation, Formal analysis, Funding acquisition, Investigation, Methodology, Project administration, Resources, Software, Validation, Writing – original draft, Writing – review & editing. **Ines R. Violante:** Data curation, Formal analysis, Software, Supervision. **Shuler Xu:** Visualization. **Toby G. Sinclair:** Investigation, Resources. **Lucia M. Li:** Data curation, Resources. **Jennifer N. Crow:** Investigation, Resources. **Marianna E. Kapsetaki:** Data curation, Resources. **Roberta Calvo:** Data curation, Project administration, Resources. **Korina Li:** Investigation, Resources. **Meenakshi Nayar:** Resources. **Nir Grossman:** Methodology, Resources, Software, Visualization. **Maneesh C. Patel:** Resources. **Richard J.S. Wise:** Conceptualization, Funding acquisition. **Paresh A. Malhotra:** Conceptualization, Funding acquisition, Investigation, Methodology, Supervision, Writing – original draft, Writing – review & editing.

## Declaration of competing interest

The authors declare that they have no known competing financial interests or personal relationships that could have appeared to influence the work reported in this paper.

## Data Availability

Data will be made available on request.
