# Supplementary material for: Targeted non-invasive brain stimulation boosts attention and modulates contralesional brain networks following right hemisphere stroke
Source: Neuroimage Clin. 2024 Mar 30;42:103599. doi: 10.1016/j.nicl.2024.103599 (PMC11019269; doi:10.1016/j.nicl.2024.103599)
Supplement: Supplementary data 1 [file mmc1.docx]

**Supplementary material**

**1**

**Inclusion and exclusion criteria**

Inclusion criteria:

- Age >18 years old;
- At least 3 months following stroke;
- Medically stable and able to attend cognitive testing;
- Evidence of attentional deficits in the acute stage.

Exclusion criteria:

- Major co-morbid conditions or pregnancy;
- Severe scalp skin lesions
- Metal implants in the head and neck (e.g., intracerebral vascular clip) or any electrically, magnetically or mechanically activated implant in the body (e.g., cardiac pacemaker);
- Likely to stop or start any psychoactive medications (i.e., antidepressants, neuroleptics) during the course of their participation in the study;
- Inability to follow instructions or complete tasks pertaining to the experiment as a result of severe cognitive deficits or language barriers;
- Inability to give informed consent;
- Claustrophobia (for MRI scanning only).

**2**

**tDCS montage**

As per the consensus-based checklist for disclosing methods for transcranial direct current stimulation concurrently to fMRI, the following specifications were used for both the behavioural and imaging study (Ekhtiari *et al.*, 2020). Direct current stimulation was delivered using two sets of battery-powered stimulators (neuroConn GmbH, Ilmenau, Germany) connected to two filter boxes (one inside and one outside the scanner room for the imaging study) via two sets of MR-compatible wires. From the filter boxes, the signal reached three disc electrodes (15mm Ø) via custom-made MR-compatible wires, so that the anode of both stimulators was combined in one electrode. Stimulators were controlled through a digital to analogue converter (DAQ) (National Instruments, Newbury, UK) receiving output from in‐house MATLAB scripts. The beginning of each stimulation session was controlled via an external trigger sent to the DAQ from a computer running a MATLAB script that specified the stimulation condition (real or sham) for a given participant, using a random order matrix set by another researcher, thus ensuring blinding of the experimenter.

Electrode location was determined by having each participant wear a flexible (size 56 or 58cm, as appropriate) EEG cap, with position sites labelled with a letter and a number according to the International 10/20 Electroencephalogram System. Four skull landmarks were measured: nasion, inion and two preauricular points. Measuring tape was applied along the midline, covering the distance from the inion to the nasion, and the position of the cap was adjusted so that the site for the vertex on the cap would be positioned halfway. The tape was reapplied transversally between the two preauricular points to find the location correctly aligned in both horizontal and vertical planes. A crayon was used to mark where the electrodes would go. The cap was then removed, and the electrodes held in place on the head using a layer of TEN20 EEG conductive paste.

**3**

**Computational modelling**

To confirm the precise current field distribution and magnitude achievable with our specific MR-compatible tDCS equipment, we performed an electromagnetic FEM simulation using Sim4Life software (Zurich MedTech AG (https://zmt.swiss/sim4life/video-training/). The model had a spatial resolution of 1x1x1mm, with anode delivering 1mA and cathodes attracting 0.50 each. This was achieved by combining field distributions from the montage with the MIDA head model, a multimodal ultra-resolution head and neck model (Iacono et al., 2015).

This computational model was set up using the following parameters:

- Three circular electrodes 1.5cm ⌀;

- 5 mm electrode thickness, considering electrode and gel layer;

- Electrodes’ location with anode over F4, cathodes over F8 and FP2 (International EEG 10-20 system).

The simulation solved the equation

$$\nabla\sigma\nabla\varphi= 0$$

where σ is the local electrical conductivity and φ is the electric potential from which the electric field and the current density can be obtained as E = - $\nabla\varphi$ and j = σ⋅ E respectively.

**4**

**Functional Imaging preprocessing and analyses**

Images were registered to high-quality brain-extracted structural T1, previously reoriented to the standard space. The tool Fsl_motion_outliers was used to detect timepoints corrupted by motion and a run was discarded if Framewise Displacement was greater than 0.5mm in more than 15% of volumes. Five patients were excluded because of excessive motion during image acquisition. Motion correction, interleaved slice timing correction, spatial smoothing (5mm Gaussian kernel filter), high-pass temporal filtering (0.01 Hz) and co-registration with T1 were then performed using MCFLIRT.

Functional imaging data were explored using Independent component analyses (ICA) for signal decomposition and Xnoiseifier (FIX) for noise removal. To isolate lesion-driven sources of variance in functional data, components that showed spatial overlap with the lesion were identified and removed.

Individual data were temporally concatenated across participants at a dimensionality of 30 components to allow identification of large-scale resting state networks common across participants. The FSL utility fslcc was used to spatially correlate each component to a set of reference networks covering most of the brain cortical/subcortical grey matter. Components showing maximum overlap with 5 large-scale networks of interest were selected for further analysis: salience network (SN), right executive control network (RECN), left executive control network (LECN), visuospatial network (VSN) and default mode network (DMN). The dual regression approach was used to obtain individual network maps. To compare conditions and groups, we performed whole-brain functional connectivity (FC) analyses, which determined the statistical dependence between each network of interest and the whole brain. Design models and contrast matrices were created using the general linear model framework. Comparisons (one-sample t- tests, paired and unpaired t-tests) were run using FSL randomise nonparametric permutation testing, with 5000 permutations and a threshold-free cluster enhancement method to control for multiple comparisons. The result was a group-level whole-brain map, with significant clusters calculated using a p<.05 threshold.

We then extracted mean connectivity strength for each network/participant/condition by applying a mask of each network to the output of dual regression. Paired and independent t-tests were used to compare mean connectivity strength between conditions, as appropriate.

Stroke-specifics: In the preprocessing stage, in order to isolate the sources of variance in functional data that were lesion-driven, components that showed spatial overlap with the lesion were removed following the method proposed by Yourganov and co-workers, whose study included chronic patients with large unilateral lesions – similar to the sample of individuals tested in the present study (Yourganov *et al.*, 2017).  The FSL MELODIC package was used to compute the Z-scored spatial maps of the independent components, which were thresholded at p < 0.05 and compared with the lesion mask for that participant. Since both the lesion mask and the thresholded independent component map were binary images, the Jaccard index (the number of voxels in the intersection divided by the number of voxels in the union) was used to quantify the amount of spatial overlap. If the Jaccard index was > 5%, the corresponding component was deemed to be significantly overlapping with the lesion mask. This step removed an additional 1-2% of data – most noise components had already been identified by FIX.

**5**

**Controlling for stimulation order**

A control analysis was performed to control for a potential contamination when real preceded sham stimulation. We included the factor ‘stimulation order’ (i.e., the chronological order of stimulation) as a co-variate in the analysis (e.g., as in Labree et al., 2020). We examined, for a group of participants who received real stimulation first, whether there was any carryover of the effect of stimulation on functional connectivity (FC) that could contaminate the sham run. Unpaired t-tests were used to compare FC within each network and between two groups of healthy participants: individuals who received real first (n=18) and those who received sham first (n=22). No significant difference was revealed for any of the networks of interest (all p values >.05).

**6**

**Clinical characteristics and performance on neurological examination**

| ID | M | SS  left | SS  bilateral | V  left | V  bilateral |
| --- | --- | --- | --- | --- | --- |
| P1 | 1/3 | 10 /10 | 0/10 | 10/10 | 10/10 |
| P2 | 1/3 | 10/10 | 10/10 | 10/10 | 10/10 |
| P3 | 3/3 | 6/10 | 3/10 | 10/10 | 0/10 |
| P4 | 0/3 | 10/10 | 7/10 | 10/10 | 10/10 |
| P5 | 0/3 | 10/10 | 10/10 | 10/10 | 10/10 |
| P6 | 1/3 | 10 /10 | 10/10 | 10/10 | 9/10 |
| P7 | 1/3 | 10/10 | 10/10 | 1/10 | 1/10 |
| P8 | 2/3 | 10 /10 | 10/10 | 10/10 | 10/10 |
| P9 | 3/3 | 9/10 | 10/10 | 10/10 | 7/10 |
| P10 | 1/3 | 10/10 | 0/10 | 10/10 | 0/10 |
| P11 | 1/3 | 8/10 | 0/10 | 10/10 | 5/10 |
| P12 | 3/3 | 0/10 | 0/10 | 10/10 | 10/10 |
| P13 | 1/3 | 10/10 | 0/10 | 9/10 | 0/10 |
| P14 | 0/3 | 10/10 | 10/10 | 10/10 | 8/10 |
| P15 | 1/3 | 10/10 | 3/10 | 5/10 | 5/10 |
| P16 | 3/3 | 10/10 | 10/10 | 0/10 | 0/10 |
| P17 | 0/3 | 7/10 | 5/10 | 10/10 | 10/10 |
| P18 | 1/3 | 10/10 | 10/10 | 10/10 | 10/10 |
| P19 | 3/3 | 10/10 | 0/10 | 10/10 | 10/10 |
| P20 | 0/3 | 10 /10 | 10/10 | 10/10 | 10/10 |
| P21 | 1/3 | 10/10 | 10/10 | 10/10 | 10/10 |
| P22 | 3/3 | 0/10 | 10/10 | 10/10 | 10/10 |

**Supplementary table 1.**

All patients presented with a first right-hemispheric stroke which caused neglect manifestations in the acute stage. M (motor upper limbs), 0=no deficit, 3=max deficit. V (visual upper quadrants) and SS (somatosensory upper limbs), 10/10= no deficit, 0/10=max deficit. This exam may have been performed the week before as part of another study, or at the beginning of the first session.

**7**

**Lesion overlay plot for right-hemispheric stroke patients**


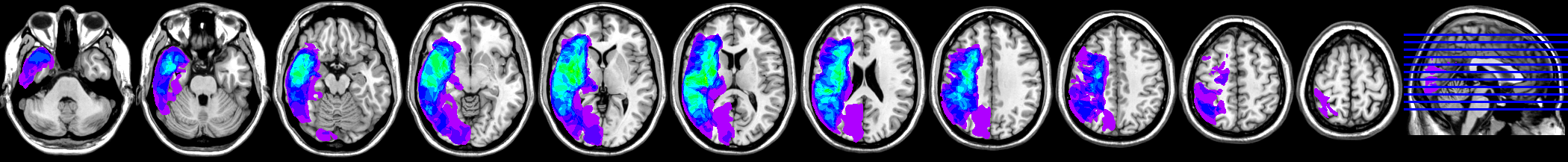


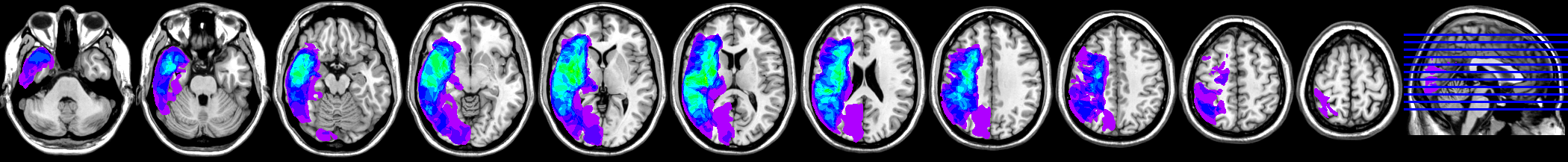


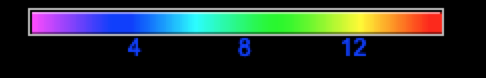


**
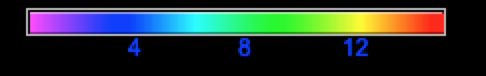
**

**Supplementary figure 1.**

Lesion overlay plot for all stroke patients who were recruited to take part in the studies following a first documented unilateral lesion in the right cerebral hemisphere.

**References**

Ekhtiari H, Ghobadi-Azbari P, Thielscher A, Antal A, Li LM, Shereen AD*, et al.* A Checklist for Assessing the Methodological Quality of Concurrent tES-fMRI Studies (ContES Checklist): A Consensus Study and Statement. medRxiv 2020: 2020.12.23.20248579.

Iacono MI, Neufeld E, Akinnagbe E, Bower K, Wolf J, Vogiatzis Oikonomidis I*, et al.* MIDA: A Multimodal Imaging-Based Detailed Anatomical Model of the Human Head and Neck. PLOS ONE 2015; 10(4): e0124126.

Yourganov G, Fridriksson J, Stark B, Rorden C. Removal of artifacts from resting-state fMRI data in stroke. NeuroImage Clinical 2017; 17: 297-305.
